# Supplementary material for: Age-related differences in stair descent balance control: Are women more prone to falls than men?
Source: PLoS One. 2021 Jan 7;16(1):e0244990. doi: 10.1371/journal.pone.0244990 (PMC7790224; doi:10.1371/journal.pone.0244990)
Supplement: Attachment — Submitted filename: Response to Editor and Reviewers.docx [file pone.0244990.s003.docx]

Dear Editor and Reviewers,

we would like to thank for your detailed and useful comments and recommendations. All changes in the manuscript are highlighted.

Below you will find our point-by-point statements to your comments. Changes are highlighted in green for Editor and in purple for Reviewer.

Yours sincerely

The authors

**EDITOR**

1) *Please improve the rationale of the abstract and remove it. For example, why are more readily seen differences between age and gender under unstable conditions?*

Done. We changed rationale as recommended.

2) *Please double check the writing on line 64 and 222.*

Sentences were rewritten.

3) *Please change hypothesize to hypothesized. - line 82*

Done.

*4)  I have two possible limitations of the study. Please addressed them.*

*I) there are fallers in the older adults sample, but not in young. This should be mentioned.
II) the number of young and older participants (especially for women) is many different. This should be mentioned too.*

Done. Mentioned limitations were added.

**REVIEWER**

*P.2, l.35-36 – I suggest starting the sentence including the word “Older women…” and change “with age” by “compared to the younger individuals” to make the sentence clearer for the readers.*

Done. Sentence was rewritten as recommended.

*P.7 Stair descent subsection – It seems that both stair descent and restabilization phases are being described in this subsection. However, there is another subsection (Restabilization) for the restabilization phase description. Please, review.*

Word "Restabilization" was replaced by "This phase".

*P.12, l.256-258 – I suggest including in this sentence the information that these data are about age comparisons, because in the way it is written, it seems that the authors are talking about gender differences.*

Done. Sentence was rewritten.
